# Differences between sectors in workers' health

**DOI:** 10.1186/2049-3258-73-S1-P25

**Published:** 2015-09-17

**Authors:** Lode Godderis, Godewina Mylle, Martijn Schouteden

**Affiliations:** 1 KULeuven-IDEWE, Leuven, Belgium; 2 IDEWE, Heverlee, Belgium

## Background

Early detection of health impairment (partly) induced by work remains difficult. Occupational Health & Safety (OHS) Providers collect a wide range of data during health surveillance. We have built a data warehouse to make OHS data available for epidemiological research.

## Aims

OHS data were analyzed to detect sector differences in registered health problems.

## Methods

Medical data were (including lifestyle indicators, categorized medication use, ICD9-CM encoded sickness absences and health complaints), collected during 1.100.000 medical examinations between 2010 and 2014, were extracted, transformed and loaded into the data warehouse. After validation and quality control, we carried out logistic regression to compare proportions between sectors (NACE) taking into account confounders like age, gender, year of examination and BMI.

## Results

The average age and seniority was respectively 39±12 and 8±9 years. Health complaints, medication use and sickness absence significantly increased with BMI and age. The proportion of employees with health problems was highest in healthcare (64%), government (61%) and manufacturing (60%) and lowest in services. In all sectors 10% of the workers reported locomotor health problems, apart from the service sector (8%). Similar results were observed for medication consumption. Neuropsychological drugs were more frequently used in healthcare workers (8%). The transport sector contained the highest proportion of cardiological medication users (12%). Finally, 30 to 59% of the employees reported at least one sickness absence. Sickness absence due to locomotor issues was highest in manufacturing (11%) and healthcare (10%), followed by government (9%) and construction (9%).

## Conclusion

This study illustrates how OHS data can be used for the detection of sector-specific trends in work related diseases. Significant differences in workers' health were observed between sectors. This information is now being used for the implementation of a sector-oriented health surveillance program.

